# Differential Role of Human Choline Kinase α and β Enzymes in Lipid Metabolism: Implications in Cancer Onset and Treatment

**DOI:** 10.1371/journal.pone.0007819

**Published:** 2009-11-12

**Authors:** David Gallego-Ortega, Ana Ramirez de Molina, Maria Angeles Ramos, Fatima Valdes-Mora, Maria Gonzalez Barderas, Jacinto Sarmentero-Estrada, Juan Carlos Lacal

**Affiliations:** 1 Translational Oncology Unit, CSIC-UAM-La Paz, Instituto de Investigaciones Biomédicas, Madrid, Spain; 2 TCD Pharma, Centro Nacional de Biotecnología, Madrid, Spain; 3 Department of Vascular Physiopathology, Hospital Nacional de Paraplejicos, SESCAM, Toledo, Spain; Fred Hutchinson Cancer Research Center, United States of America

## Abstract

**Background:**

The Kennedy pathway generates phosphocoline and phosphoethanolamine through its two branches. Choline Kinase (ChoK) is the first enzyme of the Kennedy branch of synthesis of phosphocholine, the major component of the plasma membrane. ChoK family of proteins is composed by ChoKα and ChoKβ isoforms, the first one with two different variants of splicing. Recently ChoKα has been implicated in the carcinogenic process, since it is over-expressed in a variety of human cancers. However, no evidence for a role of ChoKβ in carcinogenesis has been reported.

**Methodology/Principal Findings:**

Here we compare the *in vitro* and *in vivo* properties of ChoKα1 and ChoKβ in lipid metabolism, and their potential role in carcinogenesis. Both ChoKα1 and ChoKβ showed choline and ethanolamine kinase activities when assayed in cell extracts, though with different affinity for their substrates. However, they behave differentially when overexpressed in whole cells. Whereas ChoKβ display an ethanolamine kinase role, ChoKα1 present a dual choline/ethanolamine kinase role, suggesting the involvement of each ChoK isoform in distinct biochemical pathways under *in vivo* conditions. In addition, while overexpression of ChoKα1 is oncogenic when overexpressed in HEK293T or MDCK cells, ChoKβ overexpression is not sufficient to induce *in vitro* cell transformation nor *in vivo* tumor growth. Furthermore, a significant upregulation of ChoKα1 mRNA levels in a panel of breast and lung cancer cell lines was found, but no changes in ChoKβ mRNA levels were observed. Finally, MN58b, a previously described potent inhibitor of ChoK with *in vivo* antitumoral activity, shows more than 20-fold higher efficiency towards ChoKα1 than ChoKβ.

**Conclusion/Significance:**

This study represents the first evidence of the distinct metabolic role of ChoKα and ChoKβ isoforms, suggesting different physiological roles and implications in human carcinogenesis. These findings constitute a step forward in the design of an antitumoral strategy based on ChoK inhibition.

## Introduction

Human choline kinase alpha (ChoKα) and beta (ChoKβ) are members of the choline kinase family. In mammals this family is encoded by two separate genes, *CHKA* and *CHKB*, resulting in three different proteins with a choline/ethanolamine kinase (ChoK/EtnK) domain: ChoKα1 (NP_001268), ChoKα2 (NP_997634) and ChoKβ1 (NP_005189) [1]. ChoKα1 differs from ChoKα2 in only an extra stretch of 18 amino acids, while ChoKβ differs from ChoKα1 and ChoKα2 in approximately 40%. The presence of the ChoK/EtnK domain confers the capacity to catalyze the phosphorylation of choline (Cho) to phosphocholine (PCho) [1]. This constitutes the first step in the biosynthesis pathway of phosphatidylcholine (PC) [2]. PC is the major phospholipid in eukaryotic membranes and plays a critical role in membrane structure and also in cell signalling [2]. ChoK enzymes could be implicated also in the synthesis of phosphatidylethanolamine (PE), using as substrate ethanolamine to render phosphoethanolamine (PEtn) [1], [3]–[5].

Previous studies suggest that ChoK acts as a dimeric protein [6], [7] and the proportion of the different homo- or hetero- dimer population has been proposed to be tissue-specific [8]. Furthermore, the combination between choline kinase isoforms results in a different level of ChoK activity *in vitro* under cell-free systems conditions. Thus, the α/α homodimer is the most active choline kinase form, the β/β homodimer the less active, and the α/β heterodimer has an intermediate phenotype [8].

The specific phospholipase D-driven hydrolysis of PC generates choline and signal transduction metabolites such as PA (phosphatidic acid), and its derivatives LPA (lysophosphatidic acid) and DAG (diacylglycerol), that are important in mitogenesis and cellular transformation [9]–[11]. Choline gets further converted by ChoK into PCho, an stable metabolite able to induce mitogenesis in murine fibroblasts [12]. In addition, several oncogenes such as *RAS* or *RHOA* increase ChoKα activity resulting in higher intracellular levels of PCho [13]–[16]. Magnetic resonance spectroscopy (MRS) studies have revealed abnormal phospholipid metabolism in cancer cells [17], [18]. Furthermore, high levels of PCho have been found in tumoral cells as well as in tumour samples of cancer patients compared with the normal counterparts in breast, prostate, brain and ovarian cancer [19]–[25]. The increase in phosphoethanolamine has been also reported in transformed cells or tumor samples using MRS, although it contributes to a much lesser extent, compared with the increase of PCho, to the phosphomonoester peak [26].

The implication of ChoKα in cell growth, proliferation, initiation and progression of cancer is well documented. ChoKα is overexpressed in some of the most common cancers, such as breast, lung, colorectal, prostate [27]–[30] and bladder (unpublished observations). Furthermore, it has oncogenic activity when overexpressed in human cells [15]. Increased ChoKα mRNA levels have been recently described as an independent prognostic marker in non small cell lung cancer patients [30]. Also, human breast cancer cell lines show upregulation of ChoKα but not ChoKβ, when compared with normal mammary epithelial cells [21]. In that sense, it has been recently reported that in the prostate tumor mouse model (TRAMP), using IHQ immunodetection of ChoKβ, a low level expression of ChoKβ was found in tumoral samples compared with wild type tissues [31].

The implication of members of the Rho GTPase family in cancer onset and progression has been extensively described [32]–[35]. Evidence that ChoK is involved in malignant transformation along with Ras and RhoA has been provided [14], [15], [36]. Moreover, the activity of ChoKα is modulated by known effectors of both Ras and RhoA [15], [29].

Pharmacological inhibition of ChoKα has been proposed as a novel antitumoral strategy [2]. A strong support to this strategy is based on the use of MN58b, a well-characterized ChoKα inhibitor, which displays a potent antiproliferative effect in several tumoral cell lines in vitro, and a strong reduction of tumor growth in nude mice xenografts [14], [37]–[40].

We have compared the biochemical and biological properties of ChoKα and ChoKβ, and demonstrate that besides their homology, the latter is not able to induce cell transformation in HEK293T or MDCK cells. Moreover, we suggest differential behaviour between α and β isoforms in phospholipids metabolism. Finally, the antitumoral properties of MN58b can be attributed to its specific inhibition of ChoKα. The implications of these findings are discussed.

## Results

### Characterization of enzymatic properties of ChoKα1 and ChoKβ isoforms

**Figure 1:**
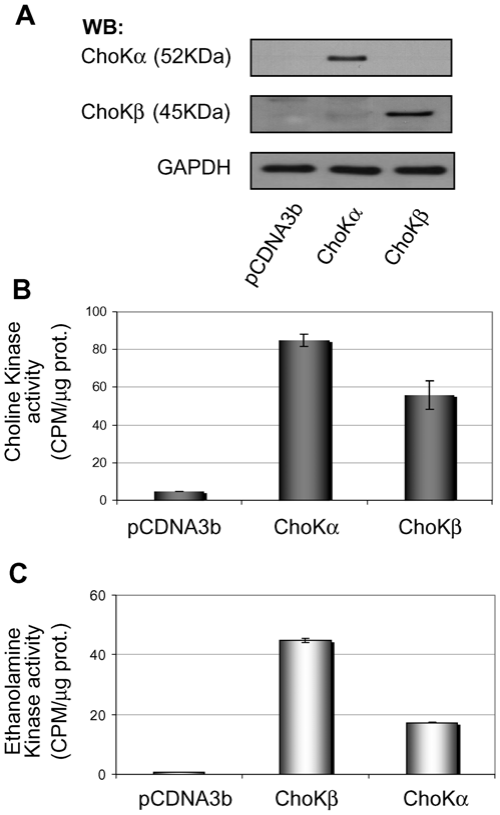
Characterization of choline and ethanolamine kinase activity of ChoKα1 and Chokβ1 in HEK293T cells. HEK293T cells were transfected with eukaryotic expression vectors of human ChoKα1 and ChoKβ1 gene. pCDNA3b empty vector was used as control. **A)** Overexpression of ChoKα1 and ChoKβ1 in HEK293T cells detected by Western Blot. GAPDH detection was used as control of expression level. **B, C)** In vitro ChoK (B) and EtnK (C) activity of choline kinase α1 and β1 isoforms in cell-free extracts of HEK293T transfected cells. Percentage of conversion of ^14^C-choline or ^14^C-ethanolamine to the phosphorylated product is represented. The experiment was performed in duplicate samples, repeated 4 times, and mean±SEM values from all experiments estimated.

The activity of both choline kinase isoforms, ChoKα1 and ChoKβ, has been described previously in different mammalian tissues, however their choline kinase and/or ethanolamine kinase activities are not fully characterized [1], [4], [41]–[43]. Thus, we first carried out a comparative analysis of the *in vitro* kinase activity of the two human ChoKα1 and ChoKβ isoforms, regarding their ability to phosphorylate choline and ethanolamine. HEK293T cells were transfected with appropriate expression vectors carrying the human CHKA or CHKB genes or an empty vector, and tested for ChoK and EtnK activity. Expression levels achieved were checked by Western Blot (Figure 1a), and the relative enzymatic activity from cell extracts estimated. Both ChoKα1 and ChoKβ displayed choline and ethanolamine kinase activities under these experimental conditions, but the rate of conversion was apparently different (Figure 1b).

We further analyze ChoK and EtnK kinetic activities for ChoKα1 and ChoKβ isoforms using the recombinant enzymes. DH5α *Escherichia coli* were transformed with the gene encoding for human ChoKα1 or ChoKβ and an enzymatic *in vitro* assay for either ChoK or EtnK activities performed. Michaelis constants (Km) for each isoform for the different substrates were obtained (Table 1). ChoKβ showed a Km for choline 2.8 times higher than ChoKα1. By contrast, the Km of ChoKβ for ethanolamine was lower than ChoKα1. These results suggest that in cell-free systems choline is a better substrate for ChoKα1 (2.85 fold) than ChoKβ, whereas ethanolamine is a better substrate for ChoKβ (5.83 fold) than ChoKα1.

**Table 1:** Michaelis constant (Km) of ChoKα and β isoforms for choline and ethanolamine.

Substrate	Isoform	Km^1^	SEM	FOLD^2^
Choline	ChoKα	0.20	0.04	1
	ChoKβ	0.57	0.08	2.85
Ethanolamine	ChoKα	12.01	2.14	5.83
	ChoKβ	2.01	0.42	1

1 Data are represented in milliMolar.

2 Referenced to the lowest Km.

Km of the different isoforms of ChoK for each substrate is indicated in each case. The results were obtained from four independent experiments using the logarithmic Michaelis-Menten formula as described in Material and Methods.

### ChoK isoforms are differentially regulated by Ras and Rho GTPases

**Figure 2:**
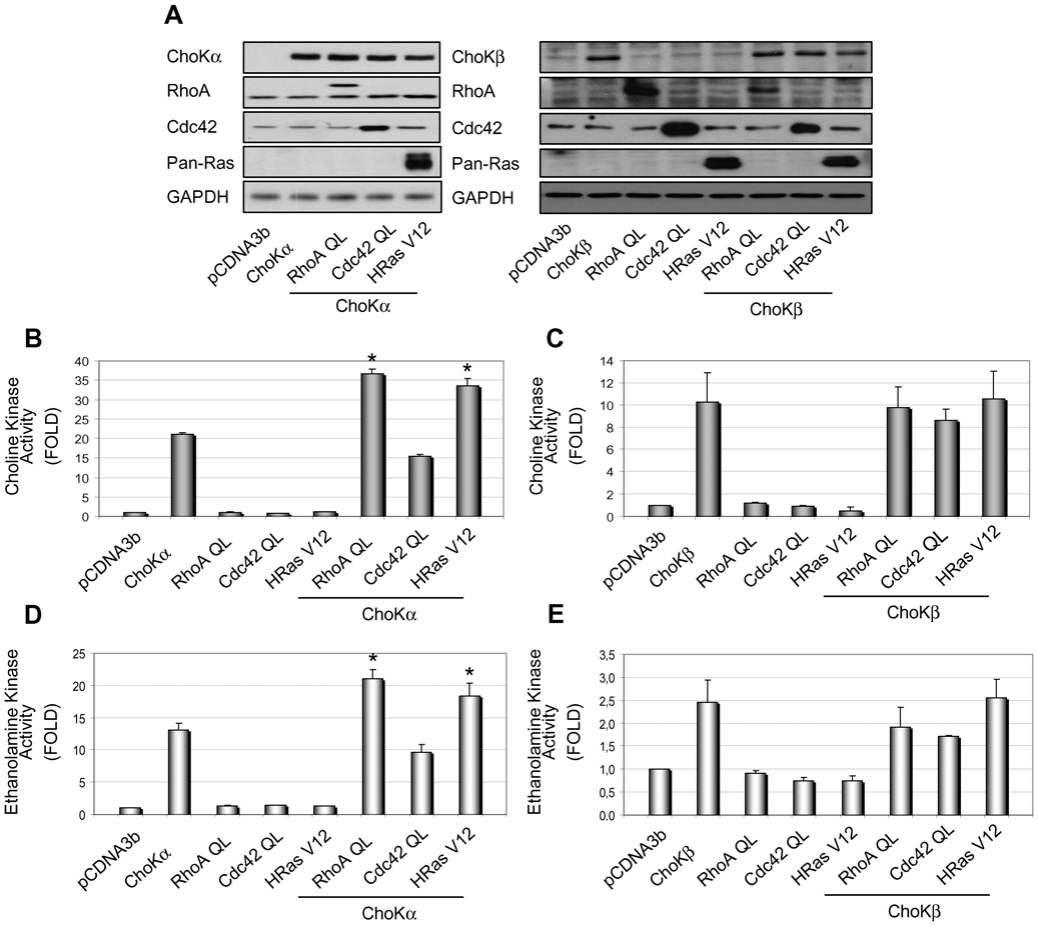
Differential activation of choline kinase α1 and β1 isoforms by Ras and Rho GTPases. Choline kinase isoforms were expressed alone or in combination with the indicated Ras and Rho GTPases and the *in vitro* ChoK activity determined. **A)** Analysis of ectopic expression by Western Blot in HEK293T transfected cell extracts of ChoKα1 (52 KDa), ChoKβ1(45 KDa), RhoA-QL(22 KDa), Cdc42-QL(25 KDa) and H-rasV12 (23 KDa). Empty vectors were used as controls for the endogenous levels, and GAPDH as loading control. **B)** and **C)** *In vitro* choline kinase activity of ChoKα1 or ChoKβ1 in the presence of enhanced expression of constitutive active forms of RhoA, Cdc42 or H-Ras. **D)** and **E)** *In vitro* ethanolamine kinase activity of ChoKα or ChoKβ in the presence of each indicated constitutive active form of GTPase. The results are represented as fold induction of conversion to the corresponding phosphorylated metabolite determined as total cpm/µg of whole cellular extract, and normalized to the empty vector transfected cells as control. Data shown represent the mean values±SEM of 3 independent experiments, each one performed with duplicate samples. Statistical significance (p≤0.05) is marked by an asterisk comparing to the activity achieved when ChoKα1 or ChoKβ1, where appropriate, are transfected alone.

Since Ras and RhoA GTPases are upstream regulators of ChoKα1 [15], [28], some of the most studied proteins of the small GTPase family including H-Ras, and the Rho-family members RhoA and Cdc42 were tested as potential upstream modulators of ChoKβ. To that end, an *in vitro* ChoK and EtnK activity assay was performed in HEK293T cells transfected with the constitutive active mutants of each GTPase (Fig. 2a) and either ChoKα1 and ChoKβ. As shown in Figure 2, none of the GTPases tested were able to significantly increase choline or ethanolamine kinase activity of ChoKβ. By contrast, under similar conditions, Ras and RhoA were able to induce a statistically significant change in the activation of both ChoK and EtnK activities of ChoKα1 (Fig. 2b and 2c).

### Differential role between ChoK isoforms in cell transformation and tumorigenesis

**Figure 3:**
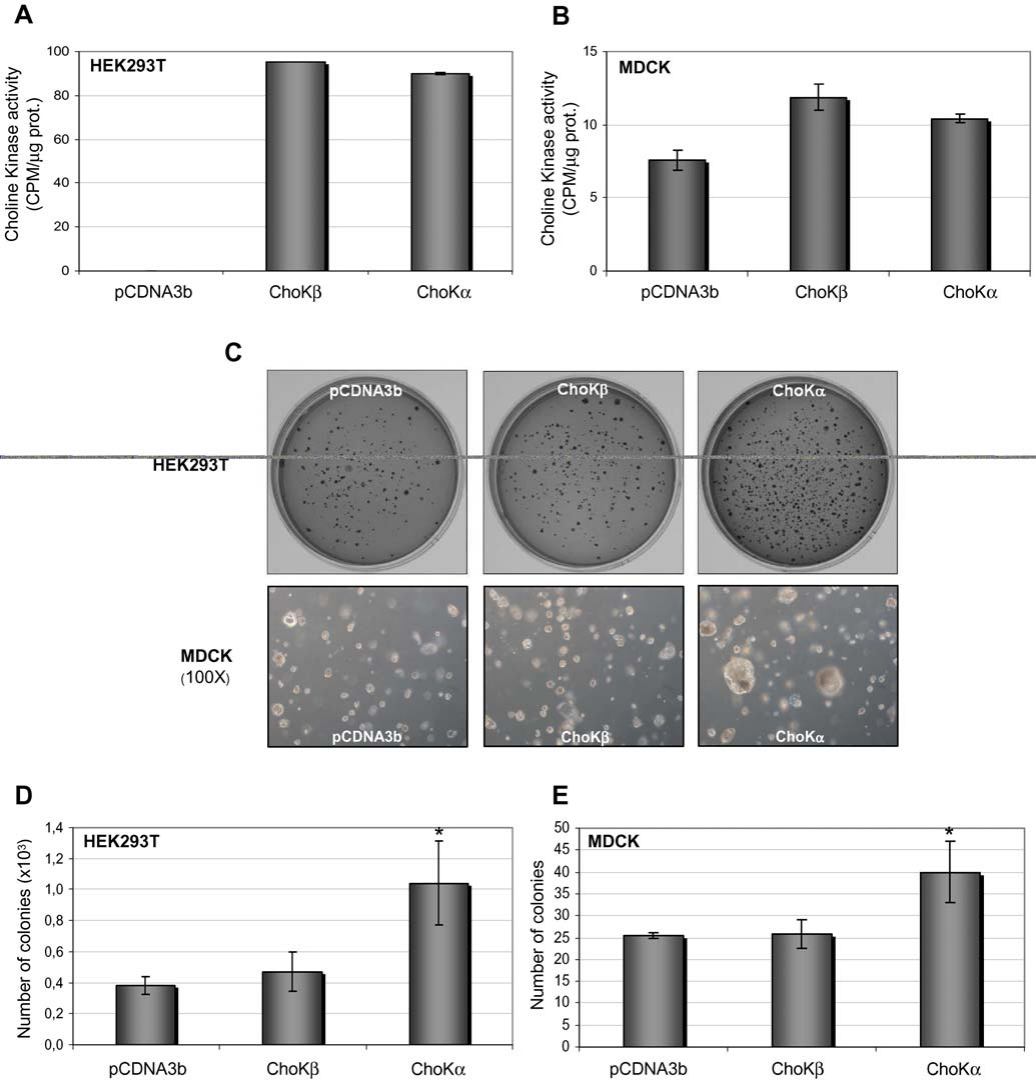
Anchorage independent cell growth of ChoKα1- and β1-overexpressing cells. **A)** and **B)** *In vitro* ChoK activity of cell-free extracts from transfected cells at the moment of plating, determined as conversion of ^14^C-labeled choline to PCho. **C)** Photographs of a representative experiment of the soft agar assay. A total of 10^5^ cells were plated per 60-mm dish, and the number of colonies quantified after 5-8 weeks of incubation. **D)** and **E)** Computer based automatic quantification of the number of colonies, mean values±SEM is represented. The assay was performed 3 independent times with triplicate samples obtaining similar results. Statistical significance (p≤0.05) is marked by an asterisk.

The implication of ChoKα1 in cell transformation and human carcinogenesis has been extensively studied, and it has been shown to display oncogenic activity [15], [36]. Due to its extensive homology, we investigated if ChoKβ could induce cellular transformation when overexpressed in non-tumorigenic cells. First, the ability of ChoKβ-transfected cells to promote anchorage independent cell growth was determined using the cell line HEK293T. As previously reported, ChoKα1 induced a significant increase in the number of soft agar colonies [15]. By contrast, under similar conditions, ChoKβ overexpression had no significant effect on the number nor the size of the colonies when compared to those generated by control, empty vector transfected HEK293T cells (Fig. 3). These results were confirmed using another non-tumorigenic cell line from a different species such as MDCK, obtaining similar results despite of the lower level of overexpression of both ChoK isoforms compared to the HEK293T cells. The differential level of expression obtained was due to the rather different efficiency in transfection for each cell lines (data not shown).

**Figure 4:**
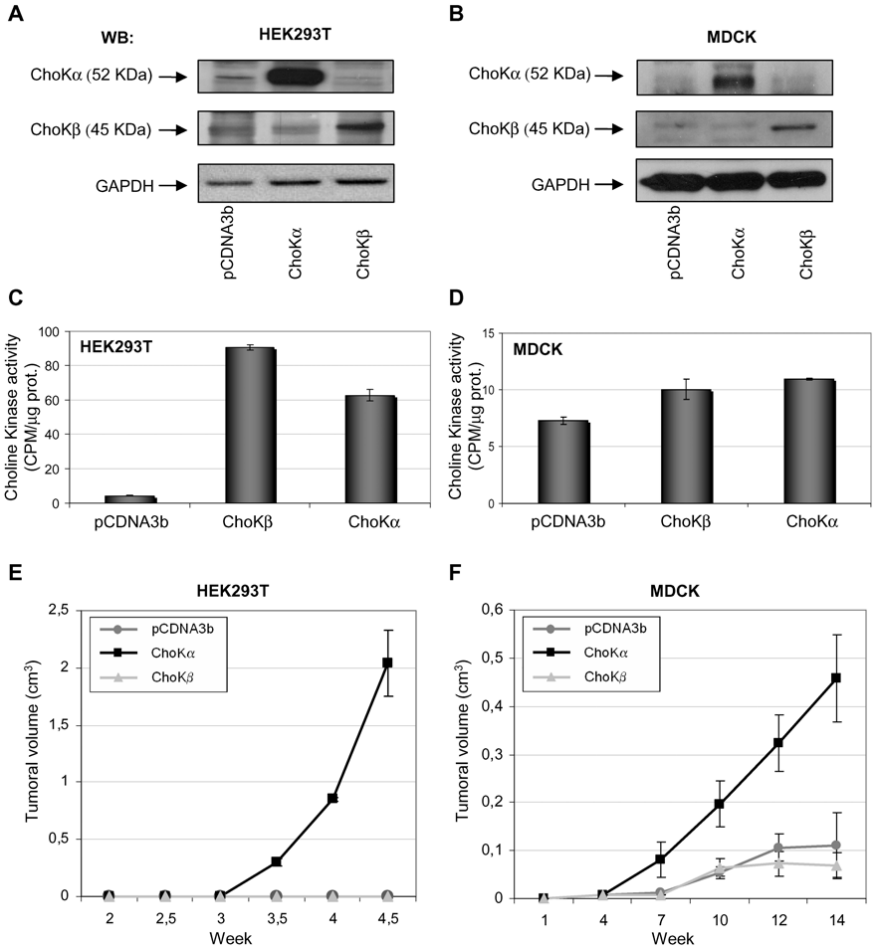
Overexpression of ChoKβ1 is not sufficient to induce tumor growth in athymic nude mice. Xenografts were established by s.c. injection of transfected HEK293T or MDCK cells in athymic nu/nu nude mice. **A)** and **B)** Western Blot analysis of ectopic expression of choline kinase isoforms in transfected HEK293T or MDCK cells, respectively, before mice inoculation. **C)** and **D)** Analysis of choline kinase activity in ChoKα1 or β1 transfected HEK293T or MDCK cells-free extracts before mice inoculation. **E)** and **F)** Volume of tumors generated by subcutaneous injection of 10^6^ transfected cells. Tumoral volume was calculated according to the formula: Vol = [D * d^2^]/2, where D and d are major and minor tumor diameters respectively. The data from HEK293T represents mean values±SEM from two independent experiments (n_1_ = 12; n_2_ = 16), the MDCK experiment correspond to an equivalent experiment with n = 12.

The ability of ChoKβ to induce tumour growth was also investigated. Both ChoKα1- and ChoKβ-transfected HEK293T or MDCK cells were subcutaneously inoculated into athymic mice. As shown in Fig. 4, the overexpression of ChoKα1 was sufficient to induce tumour growth in immunosuppressed mice in the both cell systems analyzed. By contrast, cells overexpressing ChoKβ were not able to induce tumor growth under similar conditions. As indicated above, comparison in tumor growth rates between both cell lines is related to the diverse efficiency of transfection, much higher in HEK293T (aproxm. 80–90%) than MDCK (aproxm. 15–20%). These results are in keeping with those obtained in the soft-agar experiments, and strongly indicate that overexpression of ChoKβ is not sufficient to induce tumor growth under conditions where ChoKα1 does.

### ChoKβ isoform shows preferential EtnK activity *in vivo*

**Figure 5:**
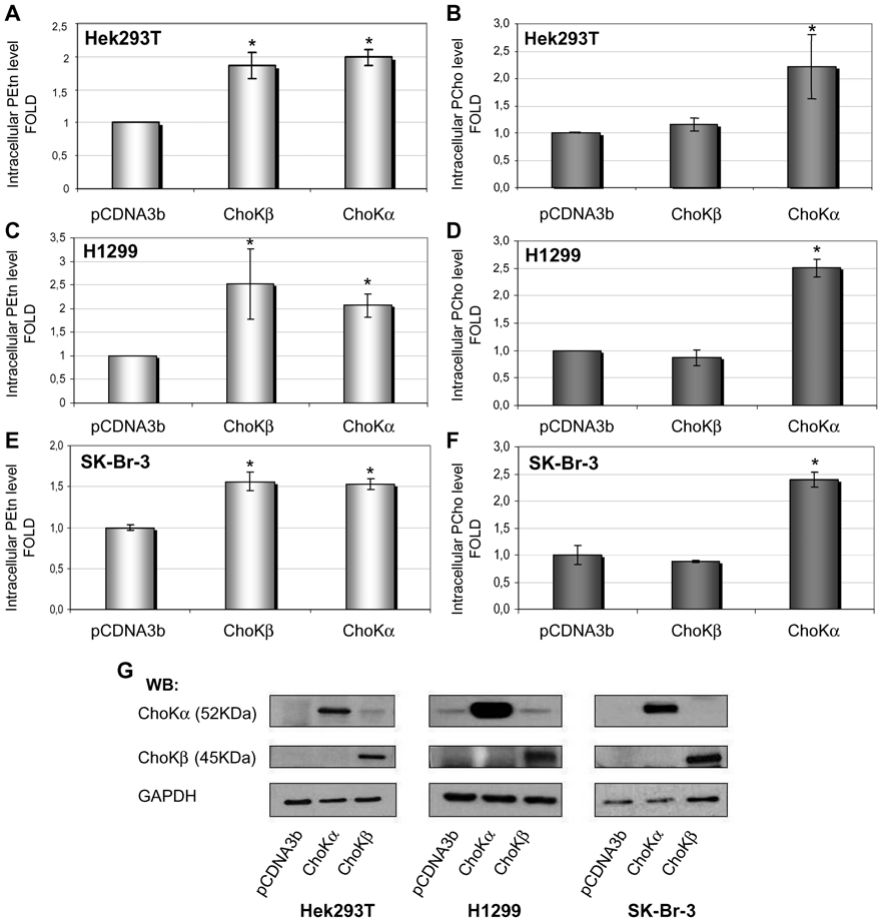
Intracellular phosphocholine and phospoethanolamine levels after ChoKα1 or ChoKβ1 transfection in human cells. HEK293T, SK-Br-3 or H1299 cells were transfected with eukaryotic expression vectors of human ChoKα1 and ChoKβ1 gene or pCDNA3b empty vector used as control. **A), C)** and **E)** Intracellular EtnK activity of choline kinase α1 and β1 isoforms in whole cells in HEK293T, H1299 and SK-Br-3 cells respectively. **B), D)** and **F)** Intracellular ChoK activity of ChoKα and ChoKβ isoforms in whole cells in HEK293T, H1299 and SK-Br-3 cells respectively. The amount of ^14^C-PCho and ^14^C-PEtn were extracted and quantified as described in Materials and Methods. The experiment was performed in triplicate samples, repeated 3 times, and mean values±SEM from all experiments estimated. Statistical significance (p≤0.05) is marked by an asterisk. A typical radio-labelling experiment results in about 70% of radioactive compound incorporation into the cells. **G)** Representative Western Blot of ectopic ChoKα1 or ChoKβ1 expression in each cell line. Baseline values chosen as 1-fold in the graphs for each cell line represent: HEK293T cells (PCho: 1153 cpm; PEtn: 1231 cpm); H1299 cells (PCho: 22821 cpm; PEtn: 13339 cpm), and SK-Br-3 cells (PCho: 18620 cpm, PEtn: 3151 cpm).

We next investigated if there was any difference in the enzymatic activity of both ChoK isoforms under *in vivo* conditions that could explain their differential biological activity. Thus, the activity of ChoKα1 and ChoKβ as ChoK and/or EtnK in a whole-cell system was tested. Intracellular lipids of human HEK293T overexpressing ChoKα1, ChoKβ or transfected with an empty vector, were extracted an analyzed. Both, the insoluble lipid fraction containing the hydrophobic lipids and total protein content were used as loading control obtaining similar results. As shown in Figure 5A, the intracellular phosphoethanolamine levels were increased to a similar extent in ChoKα1− or ChoKβ-transfected cells. However, the intracellular phosphocholine levels of ChoKβ-transfected cells were not significantly different to that of control cells, while ChoKα1-transfected cells showed an increased intracellular PCho levels (Figure 5B). Similar results were obtained using epithelial cells of different origins: human breast adenocarcinoma cells SK-Br-3 (Fig. 5C, D) or the human Non Small Cell Lung Cancer (NSCLC) cell line H1299 (Fig. 5E, F). In addition, in all cell lines analyzed, protein expression was also determined by Western Blot analysis (Fig. 5G).

Thus, the differential enzymatic activity of ChoKα1 and ChoKβ found in HEK293T is not cell line specific. These results indicate that ChoKα1 but not ChoKβ, is able to induce increased intracellular levels of PCho under *in vivo* conditions. However, under the same conditions both enzymes are able to generate phophorylethanolamine. Thus, even though ChoKβ displays both ChoK and EtnK activity under *in vitro* conditions, it shows preferential EtnK activity *in vivo*.

### Increased levels of ChoKα mRNA but not ChoKβ in tumor-derived cell lines

**Figure 6:**
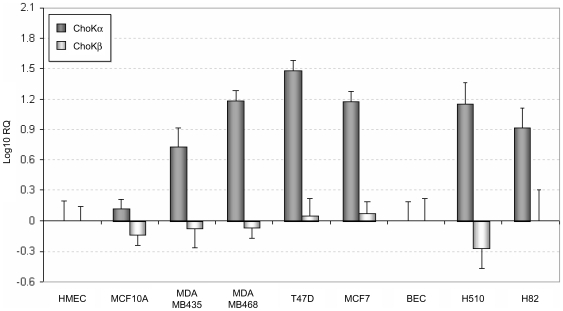
Comparative expression of ChoKα1 and ChoKβ1 mRNA in a panel of cancer cell lines. Q-PCR was performed to determine the level of expression of mRNA in non-tumorogenic mammary cell lines (HMEC, MCF10A), breast cancer cell lines (MDA-MB435, MDA-MB468, T47D, MCF7), non-tumorogenic lung cells (BEC) and lung cancer cell lines (H510, H82). The data were normalized with the endogenous 18S ribosomal RNA. For the comparison between tumoral and non-tumoral cell lines, the 2^−ΔΔCt^ method was applied and log_10_ RQ is represented. Note that the data are referred to the Human Mammary Epithelial Cells (HMEC) mRNA levels in breast cell lines and no significant difference in the level of both ChoK isoforms mRNA was found with the normal MCF10A cell line. The reference for lung cancer cells was the primary Bronchial Epithelial Cells (BEC).

In order to further investigate the relevance of each ChoK isoenzyme in tumorogenesis, we determined the levels of both ChoKα and ChoKβ mRNA in a panel of human tumour-derived cell lines by quantitative PCR technology. A panel of breast and lung cancer cell lines were compared with the primary, senescent, non-tumoral cell line HMEC (Human Mammary Epithelial Cells) or the immortalized, non-tumorogenic MCF10A cells as control breast cell lines, and primary Bronchial Epithelial Cells (BEC) as control lung cell line. All tumoral cell lines tested significantly overexpress ChoKα mRNA compared with the normal cell lines, whereas no changes were found for ChoKβ mRNA levels (Fig. 6). These results indicate that ChoKβ is not specifically upregulated in breast or lung cancer cells, suggesting that high levels of ChoKβ are not required for the promotion of cancer.

### MN58b is a specific inhibitor of ChoKα isoform

The implication of ChoKα in human carcinogenesis has been used for the design of specific inhibitors of this enzyme as a novel anticancer strategy [2], [14], [37]. MN58b is the leading compound to support this novel strategy since it has shown a potent antitumoral activity under *in vivo* conditions versus human colon, lung, breast and bladder tumor models [15], [37].

The primary structure of mammalian ChoKβ displays an overall 60% homology with that of ChoKα1 [1]. The higher degree of homology lies within the choline/ethanolamine kinase domain. This homology could make it susceptible to a similar inhibition for both ChoKα1 and ChoKβ with the same inhibitors. Thus, we have investigated the specificity of MN58b towards these two ChoK isoenzymes in order to verify if its antitumoral effect is specifically related to the inhibition of the alpha isoform, that is the one implicated in tumor progression, or the drug similarly affects both isoforms. Using the human ChoKα1 and ChoKβ recombinant proteins, we performed an *in vitro* ChoK activity inhibition assay using increasing MN58b concentrations, determining the IC_50_ for each enzyme. As expected, despite the high similarity displayed between ChoK isoforms, MN58b showed a much higher specificity against ChoKα1 (IC_50_ = 5 µM) than against ChoKβ (IC_50_ = 107.5 µM). Thus, MN58b is 21.5 times more potent against ChoKα1 than ChoKβ isoform.

## Discussion

The family of human choline/ethanolamine kinases comprises two genes, *CHKA* and *CHKB* that codify for three enzymes, ChoKα1 (52 kDa), ChoKα2 (50 kDa) and ChoKβ1 (45 kDa). ChoKα1 and ChoKα2 are almost identical, except for a stretch of 18 extra amino acids in ChoKα1, as they result from differential splicing from the same gene, *CHKA*. While the implication of ChoKα1 in the regulation of cell growth and cancer has been extensively demonstrated [13], [15], [27], [28], [37], [38], [44], preliminary evidence suggest that ChoKβ may not be involved in carcinogenesis since it is not overexpressed in breast cancer cell lines [21] nor in the TRAMP mouse prostate cancer model [31].

A distinct human gene family has been described that codifies for EtnK activity [45], suggesting that EtnK1 is the main enzyme involved in PE homeostasis. The ChoK/EtnK domain confers to ChoKα and ChoKβ the ability to function as both ChoK and EtnK activity under cell-free conditions [1], [3]–[5], but it is still unknown if these previously characterized enzymes showed any selectivity to each branch of the Kennedy pathway in the *de novo* synthesis of PC or PE. It has been recently described the different specificity towards Cho and Etn of the two isoforms of Cho/EtnK of *Tripanosoma brucei*. Whereas *Tb*Cho/Etn1 displays only EtnK activity, *Tb*Cho/Etn2 displays both ChoK and EtnK activities *in vitro* [46]. These results are in keeping with those described for murine EtnK1 that is Etn specific and EtnK2 that displays a dual ChoK/EtnK function [45], [47]. On the other hand, whereas murine Pcyt1α and Pcyt1β are involved in PC biosynthesis, Pcyt2 is focused to PE only [48].

However, it is not yet fully understood which ChoK isoform, if any, contributes *in vivo* in each pathway to maintain the normal homeostasis of both PC and PE in biological membranes. The results shown here confirm that both enzymes have the ability to phosphorylate choline and ethanolamine under cell-free conditions, either as recombinant proteins produced in *E. coli*, or in cell extracts from mammalian cells. However, we found that under whole cell conditions ChoKα1 has the ability to function as both ChoK and EtnK, but ChoKβ only affects the production of PEtn. These findings of different roles for α and β isoforms are in keeping with the information from the recently generated Knock Out (KO) mice for ChoKα and ChoKβ genes [49], [50]. Thus, ChoKβ KO mice (*rmd* mice) are viable, but develop a rostrocaudal muscular dystrophy, while normal PC lipid levels are found in most tissues analyzed except in hindlimb skeletal muscle [49]. Therefore, ChoKα is sufficient to maintain normal PC levels in most tissues. By contrast, the lack of ChoKα results in embryonic lethality, and ChoKα^+/−^ heterozygous mice display an accumulation of Cho and a reduction in PCho in liver and testis, suggesting that there is no ChoKβ compensation for PC biosynthesis *in vivo*. These results suggest different roles *in vivo* for both ChoKα and ChoKβ isoforms. Furthermore, the attenuated levels in PE found in ChoKα^+/−^ heterozygous mice suggest the involvement of ChoKα not only in the biosynthesis of PC but also in the PE pathway. This is also consistent with the fact that in ChoKβ KO mice, PE levels are unaffected, indicating that PE homeostasis is fully maintained with the EtnK1 and ChoKα proteins intact.

The group of Ishidate has provided valuable information about the *in vitro* activity of ChoK from different mouse tissues, and they have postulated that the most active form for choline kinase activity is the α/α homodimer followed by α/β heterodimers, being the β/β homodimer the least active form [8]. The *in vivo* results shown here are partially in keeping with this hypothesis. The *in vivo* activity of ChoKβ is focused in PE biosynthesis and displays higher Km for choline than ChoKα, this could be the reason why β/β dimers show low ChoK activity. When ChoKβ was overexpressed we observed an increase in the intracellular levels of PEtn but not of PCho. However no differences were found between both isoforms for the generation of PEtn, since both display similar EtnK activity.

PCho has been proposed to promote mitogenesis in mammalian cells [12]. In keeping with this, magnetic resonance spectroscopy techniques have revealed higher levels of phosphomonoesthers in tumoral samples when compared to their normal counterparts [19]–[24]. Moreover, overexpression of ChoKα1 is oncogenic [15], and enhanced ChoKα activity is a frequent feature in tumoral samples compared to normal tissues [27], [28]. Taken together all these results strongly indicate that ChoKα1 activity and PCho levels have a strong implication in cancer. Furthermore, overexpression of ChoKα1 results in an increase in EtnK activity and PEtn levels. However the latter effect by itself is not sufficient to induce cell transformation, since overexpression of ChoKβ does not induce enhanced colony formation in soft-agar or tumor growth in nude mice. These results are consistent with the hypothesis that it is the production of PCho what is linked to cell proliferation and transformation mediated by ChoK, and that the production of PEtn may not be sufficient or relevant in this process.

The above results suggest that the two ChoK isoforms investigated, besides their similarity in their primary sequences, are implicated in different metabolic pathways. Thus while ChoKα1 impinges into both PC and PE synthesis, ChoKβ affects only PE synthesis. Furthermore, the transformation capacity seems to be exclusive to the ChoKα isoform. However, since in the human HEK293T, Sk-Br-3 and H1299 cell lines, ChoKα1 overexpression produces elevated levels of both PCho and PEtn, while similar ChoKβ overexpression results only in higher levels of PEtn but normal levels of PCho, we can not rule out the possibility that cell transformation requires both ChoK and EtnK activities.

Consistent with the idea that links oncogenic activity to the function of ChoKα, but not ChoKβ, the antiproliferative and antitumoral activity of MN58b was only associated to the activity of ChoKα. Furthermore, as previously reported, the *in vivo* treatment with MN58b results in a specific decrease of PCho levels in the tumours but no significant effect on the levels of PEtn [51]. Previous results from our group have demonstrated that MN58b also inhibits choline transport [38]. However this effect has a little influence in the antiproliferative and antitumoral activity of the drug since HC-3, a much more potent inhibitor of choline transporters, is far less potent as an antiproliferative agent than MN58b [2]. Furthermore, MN58b has a differential effect on either normal or tumor cells, a strong demonstration of a differential activity due to ChoK inhibition [38]–[40]. These results are also in keeping with the observation that ChoKα but not ChoKβ is a downstream target of oncogenic molecules such as Ras and RhoA. Thus, while Ras activates ChoKα through Ral-GDS and PI3K [29], and RhoA activates ChoKα through ROCK [15], none of these oncogenic GTPases affect ChoKβ activity under similar conditions. Again, these results indicate that although both ChoK enzymes are able to phosphorylate both choline and ethanolamine under cell-free systems conditions, they display different affinities for these substrates, and in whole-cell assays conditions they are governed by distinct regulatory pathways.

Finally, the involvement of ChoKβ in breast and lung cancer has been studied, ChoKα and β mRNA levels were determined by Q-PCR. All tumour-derived cell lines assayed significantly overexpress ChoKα mRNA, while no changes were found in the expression of ChoKβ. Similar results have been recently reported, using semi-quantitative PCR in breast cancer cell lines [21]. In addition, it has been recently described that elevated mRNA levels of ChoKα is a poor prognostic factor in lung cancer [30]. These results suggest that ChoKα but not ChoKβ is playing a crucial role in human carcinogenesis.

The results shown here suggest that ChoKβ and its produced metabolites are not implicated in human cell transformation. Therefore, all the efforts aimed at dilucidating the involvement of ChoK activity in the diagnosis, prognosis and treatment of cancer have to be focused in the ChoKα isoform. In keeping with this, recently the use of specific monoclonal anti-ChoKα antibodies has been proposed as a diagnostic tool in human cancer [52]. Furthermore, the newly designed antitumoral agents are expected to be more specific and hence less toxic than the actual drugs used in conventional chemotherapy. Due to the high structural homology displayed by both choline kinase proteins, the search for new anticancer agents based on their ability to interfere with ChoK activity, must exhibit stronger antiproliferative activity based on their specificity towards the ChoKα isoform.

The lack of specific inhibition of ChoKβ by these newly designed compounds represents a new feature to take into account for the chemical improvement of ChoKα inhibitors with potential antitumoral activity. Furthermore, non-specific drugs affecting ChoKβ may result in a muscular disease produced by the lack of cell membrane lipid reparation in muscle tissue.

In addition, it has been recently demonstrated using a genetic approach that the specific inhibition of ChoKα by shRNA displays antiproliferative and antitumoral activity The high specificity of this technology provides definitive evidence of an antitumoral strategy based on ChoKα inhibition, supporting previous results with the pharmacological inhibitors. [53]–[55]

Thus, despite the high homology and similar activity displayed under cell-free conditions, ChoKα1 and ChoKβ isoforms show a different substrate ability and behave very differently under *in vivo* conditions, suggesting that in human cells, ChoKβ behaves as an EtnK and its overexpression is not able to induce higher intracellular levels of PCho. As a consequence, ChoKβ has no effect on cell proliferation and does not contribute to oncogenic transformation. Finally, ChoKα but not ChoKβ, should be used as the molecular target for the design of anticancer drugs aiming at interfering with choline kinase acitivity.

## Materials and Methods

### Ethics statement

All animals were handled in strict accordance with good animal practice as defined by the Guidelines of the Spanish Government (RD 223/1988, March 14^th^ and RD 1201/2005 October 10^th^). The animal work has been conducted under the permission and supervision of the *Consejo Superior de Investigaciones Cientificas (CSIC)* Ethic Committee as approved in the context of the appropriate Research Project.

### Cell cultures and transfections

Cells were maintained under standard culture conditions of humidity (95%), temperature (37°C) and CO_2_ (5%). Human Embryonic Kidney (HEK293T), human breast adenocarcinoma (SK-Br-3) and Canine Kidney Madin-Darby (MDCK) cells were maintained in DMEM supplemented with 10% FBS, human non-small cell lung cancer cells H1299 were maintained in RPMI with 10% FBS. HEK293T transfections were performed using calcium phosphate as previously described [56]. MDCK, SK-Br-3 and H1299 cells were transfected using lipofectamine reagent (Invitrogen, CA) following manufacturers recommendations. Eukaryotic expression vectors encoding for human ChoKα1 and β1 isoforms were pCDNA3b (Invitrogen, CA) and pLPneo (BD Biosciences Clontech, CA) respectively, both empty vectors were used as controls but only pCDNA3b is usually represented (data not shown). Constitutively activated expression pCDNA3b derivative vectors encoding for RhoA (QL), Rac1 (QL) and Cdc42 (QL) have been previously described [57]. Expression vector encoding to the constitutive active form of H-Ras (V12) have been previously described [29].

### Choline kinase *in vitro* activity assays

Eukaryotic cell extracts activity assays were carried out as previously described [14]. Cell extracts were incubated for 30 min at 37°C in a buffer containing 100 mM Tris pH 8, 10 mM MgCl_2_ and 10 mM ATP and 200 µM (55 mCi/nmol, 2 µCi/ml) of [*methyl*-^14^C] choline chloride (Amersham Biosciences, UK) for ChoK assay or 2-[^14^C]-ethan-1-ol-2-amine hydrochloride for EtnK assay (Amersham Biosciences).

For Michaelis-Menten kinetics assay, extracts of *E. coli* expressing human ChoKα1 or ChoKβ1 were used. A time course reaction was performed for each substrate, the reaction was stopped each 2 minutes and a logarithmic scale was represented to obtain the Km. Specific ChoK inhibitor MN58b was kindly provided by TCD Pharma SL (Madrid, Spain).

### Analysis of phosphocholine and phosphoethanolamine production in whole cells

HEK293T, SK-Br-3 and H1299 cells were transfected as described above. After transfection, cells were labelled to equilibrium adding 1 µCi/ml [*methyl*-^14^C] choline chloride or 1 µCi/ml 2-[^14^C]-ethan-1-ol-2-amine hydrochloride to the culture media for 24 h. Samples were resolved and quantified as previously described [37]. Briefly, cells were rinsed with PBS and fixed with 16% ice-cold trichloroacetic acid (TCA). TCA-soluble material containing choline and phosphocholine was washed three times with four volumes of diethyleter, dryed under vacuum and resuspended in water. Samples were resolved by Thin Layer Chromatography (TLC) on 60Å Silica gel plates (Whatman, NJ), using as liquid phase 0,9% NaCl: methanol: ammonium hydroxide (50∶70∶5; v/v/v). Radioactivity was automatically quantified by an electronic radiography system (Instantimager, CT). TCA insoluble material containing hydrophobic lipids was used as a loading control. The insoluble fraction was dissolved in 0,25N sodium hydroxide and total lipids were analyzed by scintillation counting.

### Anti-choline kinase β1 serum

Human ChoKβ1 gene was cloned into a prokaryotic expression vector (pGEX-4T3) (GE healthcare, UK) and expressed in DH5α *Escherichia coli*. Recombinant ChoKβ1 was then purified by the GST system (Amersham Bioscience) following manufacturer's recommendations. Purified ChoKβ1 was mixed (1∶1; v/v) with Freund's complete Adjuvant (Sigma-Aldrich, MO) and then injected i.m. into white rabbits (80 µg/rabbit). Booster injections were given every 2 weeks (60 µg/rabbit) and the sample resuspended with Freund's incomplete Adjuvant (Sigma-Aldrich) (1∶1; v/v).

### Analysis of protein expression by Western blot

Equal amounts of cell lysates were resolved by electrophoresis in a 10% SDS-PAGE. ChoKα expression levels were detected using monoclonal antibody AD3 as previously described [52]. ChoKβ1 was detected using the polyclonal antiserum described above as primary antibody at a standard dilution of 1/5000. GTPases antibodies: RhoA (Santa Cruz Biotechnology, Germany), Rac1 (Upstate, NY), pan-Ras (Santa Cruz Biotechnology), Cdc42 (BD Biosciences, transduction labs) and GAPDH antibody (Chemicon International, CA) were used following manufacturers recommendations. HRP-conjugated secondary antibodies were from Santa Cruz Biotechnology. The specific binding was detected using the chemioluminiscence detection kit ECL (Amersham Bioscience) following the manufacturer indications.

### Soft agar anchorage-independent growth and *in vivo* tumorigenic assays

Anchorage-independent growth assays were performed at 5–6 weeks of incubation in HEK293T and 6–10 weeks in MDCK, as previously described [15], plating 10^5^ cells per 60-mm dish. Crystal violet staining was performed to enhance colony contrast. The number of colonies was automatically quantified by computer based image software (Image J). For the *in vivo* assays, cells were injected (10^6^ cells per mouse flank) s.c. in *nu/nu* athymic mice. Mice were kept under standard laboratory conditions according to the guidelines of Spanish Government.

### Quantitative PCR

ChoKα1 or β1 mRNA levels were quantified by real-time reverse transcriptase PCR. The RNA was extracted from the cell lines using QIAshredder following the RNeasy Mini kit (Qiagen, Inc.) according to the manufacturer's instructions. The amount of 0.9 µg of total RNA, in a final concentration of 10 ng/µl per reaction, was retro transcribed by High-Capacity cDNA Archive Kit (Applied Biosystems) to the cDNA preparation. The reverse transcription conditions were 25°C for 10 min and 37°C for 2 h. Then, each cDNA sample was analyzed in triplicate using the ABI PRISM 7700 Sequence Detector (PE Applied Biosystems). Real-time PCR was carried out using Taqman Universal PCR Master Mix (Applied Biosystems), containing ROX to normalize emissions. Primers used for amplification of ChoKα, ChoKβ and 18S ribosomal RNA were purchased from Applied Biosystems as Taqman Gene Expression Assays (ChoKα1 ID: Hs00608045_m1, ChoKβ1 ID: Hs01925200_s1 and 18S ribosomal RNA ID: Hs99999901_s1). For thermal cycling, the following conditions were applied: 10 min at 95°C and 40 cycles of 15 sec at 95°C and 1 min at 60°C. The data are presented as Log_10_ RQ (Relative Quantity).

### Statistical analysis

Mean comparisons of ChoK activity or in-cell PCho increase between different groups were performed. Continuous variables with normal distribution were compared by T-test and non-normal distribution variables were compared by means of the Kruskal Wallis and Mann-Whitney U tests. Statistical significance was defined as p≤0.05. The statistical analyses were performed using SPSS software, version 13.0 (Inc, Chicago, Illinois). The method used to analyze data from real-time PCR experiments was 2^−ΔΔCt^ method [58], comparing the relative gene expression between normal and tumoral cell lines normalized to 18S ribosomal RNA as endogenous reference gene.
